# Dronedarone-Mediated Acute Hepatic and Renal Toxicity

**DOI:** 10.7759/cureus.9611

**Published:** 2020-08-07

**Authors:** Zarak H Khan, Kashif Mukhtar, Munis M Ahmed, Syeda Ramsha Zaidi, Randa Abd Algayoum

**Affiliations:** 1 Internal Medicine, St. Mary Mercy Hospital, Livonia, USA; 2 Internal Medicine, King Edward Medical University, Lahore, PAK

**Keywords:** dronedarone, acute liver failure, acute renal injury

## Abstract

Dronedarone, a drug similar in chemical properties to amiodarone, was designed to have similar pharmacodynamic properties as amiodarone with fewer side effects. Although there have been cases of chronic liver enzyme elevation with dronedarone, only a few cases have been reported in literature where it has led to rapid onset of liver failure. We present the case of an 86-year-old male who developed acute liver failure concomitantly with acute kidney failure after he was started on dronedarone therapy.

## Introduction

Dronedarone is a rhythm-control agent with chemical properties similar to amiodarone. The drug was developed to have similar anti-arrhythmic effects as amiodarone but without most of its adverse effects [1]. It is frequently used for controlling rhythm in patients with atrial fibrillation (AF) [2]. Several clinical trials have shown that the drug has been associated with mild liver enzymes elevations in up to 12% of patients [3]. However, there have only been two reported cases of acute liver failure in literature that occurred after the administration of dronedarone. Besides affecting the hepatic system, several adverse drug reaction reports have also described cases of dronedarone-induced acute renal failure (ARF) [4]. The case we present here has been previously presented as a poster presentation at the American College of Gastroenterology Conference in October 2019 [5].

## Case presentation

**Figure 1:**
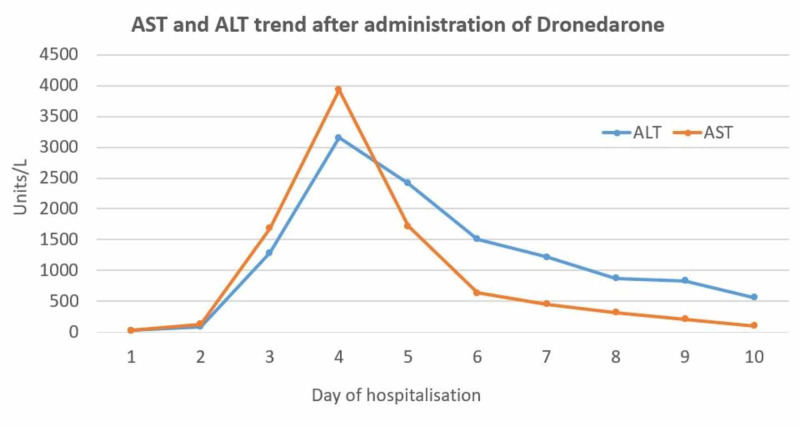
AST and ALT trend over the course of hospitalization. AST, aspartate aminotransferase; ALT, alanine aminotransferase

**Figure 2:**
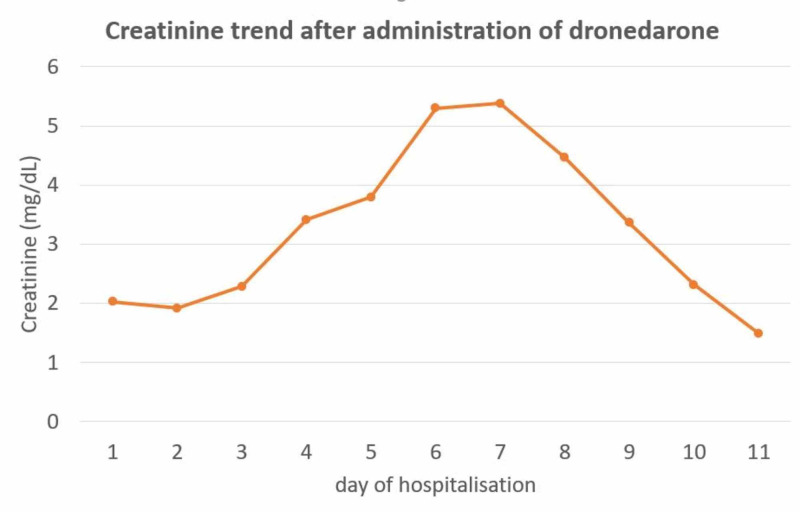
Creatinine trend over the course of hospitalization.

An 86-year-old male with a past medical history of AF on amiodarone, chronic obstructive pulmonary disease, chronic kidney disease stage 3, and primary hypertension presented with a one-day duration of dyspnea and palpitations. On examination, the patient was found to be in AF. Electrocardiogram (EKG) showed a rapid ventricular response and a heart rate of 127 beats/min. Vitals were otherwise stable and the cardiovascular exam revealed irregular heart rate. Laboratory values on admission were significant for creatinine of 2.02 (patient’s baseline was around 1.60). In the ED, the patient was initially started on diltiazem drip which was later discontinued due to an IV site reaction. He was then started on dronedarone for rhythm control on the recommendation of the cardiologist service. The patient was given three doses of dronedarone. However, his heart rate did not significantly decrease; therefore, electrical cardioversion was performed. The patient’s liver function tests, taken 24 hours after the last dose of dronedarone and post cardioversion, showed elevated alanine aminotransferase (ALT) of 3155 IU/L, aspartate aminotransferase (AST) level of 3930 IU/L, alkaline phosphatase of 144 IU/L, total bilirubin of 2.4 µmol/L, and direct bilirubin of 1.0 µmol/L (Figure 1). International normalized ratio (INR) was also elevated to 6.1. Viral hepatitis panel was ordered which was negative. The patient's clinical course was further complicated by ARF with creatinine rising up to 5.38 mg/dL from 2.02 mg/dL at admission (Figure 2). He developed worsening fatigue at this time. It was suspected that the patient developed hepatic and renal injury due to dronedarone administration; therefore, the drug was discontinued and the patient was started on supportive measures. During the next four days the patient’s laboratory values including liver function tests and creatinine trended down and his symptoms of lethargy and weakness improved. At outpatient follow up, it was seen that the patient had complete resolution of renal and hepatic functions.

## Discussion

Dronedarone is an anti-arrhythmic drug used for rhythm control in patients with AF. It was initially developed with the goal of providing similar efficacy to amiodarone and was approved by the FDA in 2009 [6]. As dronedarone lacks iodine it was thought that the drug will have lower adverse effects on the thyroid gland and possibly liver than amiodarone [7]. Despite the promising results from initial clinical trials, the drug is only used in well-selected patients due to significant side effects and possibly increased risk of mortality and hospitalization in patients with advanced NYHA class and permanent AF [6].

Mild elevation of hepatic enzymes with dronedarone administration has been well established. In the Euridis/Adnois trials, it was found that about 12% of patients who received dronedarone had developed elevated liver enzymes [8]. This elevation in transaminases was a gradual phenomenon rather than an acute one. Also, in most of these cases, the withdrawal of dronedarone therapy led to the normalization of liver function tests.

There have been only a few cases described in literature where patients developed acute liver failure due to dronedarone administration. The first case occurred in a 70-year-old woman who had been on dronedarone therapy for six months due to AF. Her liver injury was severe enough to cause hepatic encephalopathy requiring liver transplantation [9]. Another case occurred in an 83-year-old African American lady who presented with symptomatic tachycardia secondary to AF. She developed elevated liver enzymes with AST of >1600 U/L, ALT of 1791 U/L, alkaline phosphatase of 208 U/L, INR of 1.79, and albumin of 2.5 g/dL. However, unlike the previous case, her liver enzymes normalized within five days after cessation of the dronedarone therapy and with supportive care [10]. Similarly, the patient in our case also had a sudden severe derangement of liver enzymes which returned to baseline within a few days after discontinuation of therapy (Figure 1).

There have been several cases of acute renal impairment associated with dronedarone as well. Young et al. presented a case of acute kidney injury in a 71-year-old male five days after starting the drug. The patient required hemodialysis for a few days after which his renal function improved in two to three weeks [11]. Another similar case has been described by Coons et al. where the patient developed ARF and worsening liver failure due to dronedarone therapy [12]. This is the only case which has described worsening liver failure with concurrent acute kidney injury. The patient in our case also developed both hepatic and renal failure at the same time without any other etiology to explain these findings.

## Conclusions

Our case adds to the growing literature of acute hepatic and renal dysfunction secondary to dronedarone. Physicians should be aware of these side effects before initiating the patient on dronedarone therapy. Routine laboratory studies including liver function tests (LFTs) and creatinine should be ordered on these patients. In the case where the patient develops a sudden elevation in liver enzymes or creatinine, the drug should be immediately discontinued and serial laboratory monitoring should be done until the values have returned to baseline.
